# Efficacy of Capecitabine and Temozolomide Regimen in Neuroendocrine Tumors: Data From the Turkish Oncology Group

**DOI:** 10.1093/oncolo/oyad257

**Published:** 2023-09-07

**Authors:** Çağlar Ünal, Abdulmunir Azizy, Senem Karabulut, Didem Taştekin, Arif Akyıldız, Serkan Yaşar, Şuayib Yalçın, Eyüp Çoban, Türkkan Evrensel, Ziya Kalkan, Zeynep Oruç, Sümeyra Derin, Zeynep Hande Turna, Doğan Bayram, Fahriye Tuğba Köş, Mehmet Ali Nihat Şendur, Nadiye Sever, Özlem Ercelep, Mustafa Seyyar, Umut Kefeli, Kazım Uygun, Melike Özçelik, Sercan Ön, Ulus Ali Şanlı, Kübra Canaslan, İlkay Tuba Ünek, Kadriye Bir Yücel, Nuriye Özdemir, Ozan Yazıcı, Halil Göksel Güzel, Derya Kıvrak Salim, Sema Sezgin Göksu, Ali Murat Tatlı, Çetin Ordu, Oğuzhan Selvi, Abdullah Sakin, Mehmet Emin Büyükbayram, Bengü Dursun, Yüksel Ürün, Hacı Arak, Gözde Ağdaş, Muzaffer Uğraklı, Engin Hendem, Melek Karakurt Eryılmaz, Burak Bilgin, Atakan Topçu, Melih Şimşek, Mahmut Büyükşimşek, Büşra Akay, Gülçin Şahingöz Erdal, Fatih Karataş, Özkan Alan, Melek Çağlayan, Fatma Akdağ Kahvecioğlu, Ayşe Demirci, Nail Paksoy, Bülent Çetin, Mahmut Gümüş, Naziye Ak, Yasemin Aydınalp, Semra Paydaş, Deniz Can Güven, Saadettin Kılıçkap, Sezer Sağlam

**Affiliations:** Division of Medical Oncology, Department of Internal Medicine, Kartal Dr. Lütfi Kırdar City Hospital, İstanbul, Turkey; Division of Medical Oncology, Department of Internal Medicine, İstanbul University Institute of Oncology, İstanbul, Turkey; Division of Medical Oncology, Department of Internal Medicine, İstanbul University Institute of Oncology, İstanbul, Turkey; Division of Medical Oncology, Department of Internal Medicine, İstanbul University Institute of Oncology, İstanbul, Turkey; Division of Medical Oncology, Department of Internal Medicine, Hacettepe University Institute of Oncology, Ankara, Turkey; Division of Medical Oncology, Department of Internal Medicine, Hacettepe University Institute of Oncology, Ankara, Turkey; Division of Medical Oncology, Department of Internal Medicine, Hacettepe University Institute of Oncology, Ankara, Turkey; Division of Medical Oncology, Department of Internal Medicine, Uludağ University Hospital, Bursa, Turkey; Division of Medical Oncology, Department of Internal Medicine, Uludağ University Hospital, Bursa, Turkey; Division of Medical Oncology, Department of Internal Medicine, Dicle University Hospital, Diyarbakır, Turkey; Division of Medical Oncology, Department of Internal Medicine, Dicle University Hospital, Diyarbakır, Turkey; Division of Medical Oncology, Department of Internal Medicine, Cerrahpaşa University Hospital, İstanbul, Turkey; Division of Medical Oncology, Department of Internal Medicine, Cerrahpaşa University Hospital, İstanbul, Turkey; Division of Medical Oncology, Department of Internal Medicine, Ankara City Hospital, Ankara, Turkey; Division of Medical Oncology, Department of Internal Medicine, Ankara City Hospital, Ankara, Turkey; Division of Medical Oncology, Department of Internal Medicine, Ankara City Hospital, Ankara, Turkey; Division of Medical Oncology, Department of Internal Medicine, Marmara University Hospital, İstanbul, Turkey; Division of Medical Oncology, Department of Internal Medicine, Marmara University Hospital, İstanbul, Turkey; Division of Medical Oncology, Department of Internal Medicine, Kocaeli University Hospital, Kocaeli, Turkey; Division of Medical Oncology, Department of Internal Medicine, Kocaeli University Hospital, Kocaeli, Turkey; Division of Medical Oncology, Department of Internal Medicine, Kocaeli University Hospital, Kocaeli, Turkey; Division of Medical Oncology, Department of Internal Medicine, Ümraniye Research and Training Hospital, İstanbul, Turkey; Division of Medical Oncology, Department of Internal Medicine, Ege University Hospital, İzmir, Turkey; Division of Medical Oncology, Department of Internal Medicine, Ege University Hospital, İzmir, Turkey; Division of Medical Oncology, Department of Internal Medicine, Dokuz Eylül University Hospital, İzmir, Turkey; Division of Medical Oncology, Department of Internal Medicine, Dokuz Eylül University Hospital, İzmir, Turkey; Division of Medical Oncology, Department of Internal Medicine, Gazi University Hospital, Ankara, Turkey; Division of Medical Oncology, Department of Internal Medicine, Gazi University Hospital, Ankara, Turkey; Division of Medical Oncology, Department of Internal Medicine, Gazi University Hospital, Ankara, Turkey; Division of Medical Oncology, Department of Internal Medicine, Antalya Research and Training Hospital, Antalya, Turkey; Division of Medical Oncology, Department of Internal Medicine, Antalya Research and Training Hospital, Antalya, Turkey; Division of Medical Oncology, Department of Internal Medicine, Akdeniz University Hospital, Antalya, Turkey; Division of Medical Oncology, Department of Internal Medicine, Akdeniz University Hospital, Antalya, Turkey; Division of Medical Oncology, Department of Internal Medicine, Gayrettepe Florence Nightinagle Hospital, İstanbul, Turkey; Division of Medical Oncology, Department of Internal Medicine, Okmeydanı Research and Training Hospital, İstanbul, Turkey; Division of Medical Oncology, Department of Internal Medicine, Bahçelievler Medipol Hospital, İstanbul, Turkey; Division of Medical Oncology, Department of Internal Medicine, Ataturk University Hospital, Erzurum, Turkey; Division of Medical Oncology, Department of Internal Medicine, Ankara University Hospital, Ankara, Turkey; Division of Medical Oncology, Department of Internal Medicine, Ankara University Hospital, Ankara, Turkey; Division of Medical Oncology, Department of Internal Medicine, Gaziantep University Hospital, Gaziantep, Turkey; Division of Medical Oncology, Department of Internal Medicine, Osmangazi University Hospital, Eskişehir, Turkey; Division of Medical Oncology, Department of Internal Medicine, Konya Meram University Hospital, Konya, Turkey; Division of Medical Oncology, Department of Internal Medicine, Konya Meram University Hospital, Konya, Turkey; Division of Medical Oncology, Department of Internal Medicine, Konya Meram University Hospital, Konya, Turkey; Division of Medical Oncology, Department of Internal Medicine, Konya City Hospital, Konya, Turkey; Division of Medical Oncology, Department of Internal Medicine, Bezmialem Vakıf University Hospital, İstanbul, Turkey; Division of Medical Oncology, Department of Internal Medicine, Bezmialem Vakıf University Hospital, İstanbul, Turkey; Division of Medical Oncology, Department of Internal Medicine, Adana Research and Training Hospital, Adana, Turkey; Division of Medical Oncology, Department of Internal Medicine, Ankara Yurtaslan City Hospital, Ankara, Turkey; Division of Medical Oncology, Department of Internal Medicine, Bakırköy Sadi Konuk Research and Training Hospital, İstanbul, Turkey; Division of Medical Oncology, Department of Internal Medicine, Karabük University Hospital, Karabük, Turkey; Division of Medical Oncology, Department of Internal Medicine, Koc University Hospital, İstanbul, Turkey; Division of Medical Oncology, Department of Internal Medicine, Konya Selçuk University Hospital, Konya, Turkey; Division of Medical Oncology, Department of Internal Medicine, Sakarya Research and Training Hospital, Sakarya, Turkey; Division of Medical Oncology, Department of Internal Medicine, Sakarya Research and Training Hospital, Sakarya, Turkey; Division of Medical Oncology, Department of Internal Medicine, Tekirdağ City Hospital, Tekirdağ, Turkey; Division of Medical Oncology, Department of Internal Medicine, Ondokuz Mayıs University Hospital, Samsun, Turkey; Division of Medical Oncology, Department of Internal Medicine, Göztepe Medeniyet University Hospital, İstanbul, Turkey; Division of Medical Oncology, Department of Internal Medicine, İstanbul Florence Nightinagle Hospital, İstanbul, Turkey; Division of Medical Oncology, Department of Internal Medicine, Adana Balcalı University Hospital, Adana, Turkey; Division of Medical Oncology, Department of Internal Medicine, Adana Balcalı University Hospital, Adana, Turkey; Division of Medical Oncology, Department of Internal Medicine, Elazığ Fethi Sekin City Hospital, Elazığ, Turkey; Division of Medical Oncology, Department of Internal Medicine, Ankara Liv Hospital, Ankara, Turkey; Division of Medical Oncology, Department of Internal Medicine, Demiroglu Bilim University, İstanbul, Turkey

**Keywords:** capecitabine, CAPTEM, neuroendocrine neoplasia, neuroendocrine tumors, temozolomide

## Abstract

**Introduction:**

This study aims to report the efficacy and safety of capecitabine plus temozolomide (CAPTEM) across different lines of treatment in patients with metastatic neuroendocrine tumors (NETs).

**Methods:**

We conducted a multicenter retrospective study analyzing the data of 308 patients with metastatic NETs treated with CAPTEM between 2010 and 2022 in 34 different hospitals across various regions of Turkey.

**Results:**

The median follow-up time was 41.0 months (range: 1.7-212.1), and the median age was 53 years (range: 22-79). Our results across the entire patient cohort showed a median progression-free survival (PFS) of 10.6 months and a median overall survival (OS) of 60.4 months. First-line CAPTEM treatment appeared more effective, with a median PFS of 16.1 months and a median OS of 105.8 months (median PFS 16.1, 7.9, and 9.6 months in first-, second- and ≥third-line respectively, *P* = .01; with median OS values of 105.8, 47.2, and 24.1 months, respectively, *P* = .003) In terms of ORR, the first-line treatment again performed better, resulting in an ORR of 54.7% compared to 33.3% and 30.0% in the second and third or higher lines, respectively (*P* < .001). Grade 3-4 side effects occurred only in 22.5% of the patients, leading to a discontinuation rate of 9.5%. Despite the differences in outcomes based on treatment line, we did not observe a significant difference in terms of side effects between the first and subsequent lines of treatment.

**Conclusions and Relevance:**

The substantial superior outcomes in patients receiving first-line CAPTEM treatment highlight its potential as an effective treatment strategy for patients with metastatic NET.

## Teaser

This article reports on the efficacy and safety of capecitabine plus temozolomide (CAPTEM) across different lines of treatment in patients with metastatic neuroendocrine tumors.

**Implications for Practice**

This is a large multicenter study evaluating the CAPTEM regimen reporting results in the largest patient cohort to date (*n* = 308 patients, 34 different hospitals). We found that first-line CAPTEM treatment yielded higher ORR, PFS, and OS benefits compared to other lines of treatment regardless of tumor location. We report CAPTEM as a relatively safe treatment for patients with metastatic neuroendocrine tumors.

## Introduction

Neuroendocrine neoplasms (NENs) represent a diverse group of tumors, the incidence of which has surged 7-fold in the past 40 years.^1,2^ Many neuroendocrine tumors (NETs) are asymptomatic and nonfunctioning, often discovered incidentally at autopsy series. Alternatively, diagnosis is made retrospectively following an appendectomy or liver biopsy.^3^ These neoplasms most frequently originate in the digestive system and, to a lesser extent, in the lungs.^4^ NENs are frequently detected in the metastatic stage, and their clinical behavior and prognosis vary greatly according to the primary tumor location, tumor morphology, grade, and Ki-67 level.^5^ For patients with metastatic or relapsed disease, a multidisciplinary approach is essential. Treatment options, depending on the extent of the disease, may include peptide receptor radionuclide therapy (PRRT), somatostatin analogs, targeted therapies (everolimus, sunitinib), or temozolomide-based chemotherapy regimens. These options are determined at the clinician’s discretion.^6^

Temozolomide, a less toxic oral derivative of intravenous dacarbazine, is an alkylating agent that induces cell apoptosis through DNA methylation. As this effect is not cell cycle-specific, it can influence every stage of tumor growth, including slow-growing, low-grade tumors.^7^ Temozolomide, recognized for its activity against glioblastoma, melanoma, and NETs, can be administered alone or combined with other agents such as capecitabine, bevacizumab, or everolimus.^8^ Capecitabine, an oral chemotherapeutic prodrug, is enzymatically converted to 5-fluorouracil (5-FU), thereby acting as an antimetabolite in tumor tissue.^9^ Clinical studies investigating its use in NETs are increasing.^10,11^ Despite the efficacy of the combined temozolomide and capecitabine regimen in patients with NETs, the mechanism underlying this activity remains unclear.^12^ The antimetabolite action of capecitabine involves incorporating 5-fluorodeoxyuridine triphosphate into DNA. By inhibiting thymidylate synthetase, it reduces MGMT (O^6^-methylguanine–DNA methyltransferase) repair activity, leading to decreased thymidine levels. This reduction in MGMT activity can enhance the effectiveness of temozolomide treatment, while a high MGMT level may reduce sensitivity to 5-FU.^13^ The role of MGMT remains controversial in the literature, leading to current recommendations against routine MGMT testing.^14,15^

In this study, our objective was to examine the efficacy and side-effect profile of the CAPTEM regimen in patients with metastatic neuroendocrine tumors.

## Materials and Methods

We conducted a multicenter retrospective study analyzing the data of 308 patients with de novo or relapsed metastatic stage NETs. These patients were treated with CAPTEM between 2010 and 2022 across 34 different hospitals in various regions of Turkey. To examine as homogeneous a cohort as possible, we included patients with confirmed NETs. Patients with grades 1 and 2 tumors, with unknown Ki-67 levels, were included. In addition, we included 14 patients with tumors of unknown grade that had Ki-67 levels between 1% and 20%. No patient had a Ki-67 level above 55%.

Patients were grouped according to the line of treatment when CAPTEM was administered (first, second, ≥third), Ki-67 level, tumor grade, and the location of the primary tumor. Patients had to have received at least one cycle of CAPTEM treatment and have radiologically measurable or evaluable metastatic disease according to Response Evaluation Criteria in Solid Tumors (RECIST) 1.1 criteria.

Specialized pathologists evaluated paraffin-embedded and formalin-fixed tissues obtained from the primary tumor or metastatic lesion in each hospital’s pathology unit. Prior therapies with somatostatin analogs, PRRT, 5-FU-based regimens, everolimus, or sunitinib were permitted. Patients who had received local treatments such as transarterial radioembolization (TARE), transarterial chemoembolization (TACE), or radiofrequency ablation (RFA) were also included. Eleven patients who either received one drug in the CAPTEM regimen without using the other or failed to follow up regularly were excluded. The study included patients who received at least one cycle of capecitabine 750 mg/m^2^ PO BID on days 1-14 and temozolomide 150-200 mg/m^2^ PO QD on days 10-14 repeated every 28 days. Patients often received somatostatin analogs concurrently with first-line CAPTEM regimen. In addition, concomitant use of somatostatin analog was allowed in other lines of CAPTEM regimen. Patients who completed a minimum of 6 cycles of CAPTEM without progression and continued with monthly intramuscular somatostatin analog therapy intramuscularly as maintenance therapy until progression were also included. MGMT status was not routinely evaluated.

Tumor response to treatment was assessed every 3-4 months. We collected data on the duration of CAPTEM administration, the reasons for discontinuation of the treatment, and side effects. Data entry included the objective response rate (ORR), which included patients with complete response (CR) or partial response (PR). The determinations of complete response (CR), partial response (PR), and stable disease (SD) were primarily based on the radiology reports provided by our clinical radiologists. They routinely include measurements of tumors in their reports, and for our study, we utilized these measurements to categorize responses according to RECIST v1.1.

We calculated the disease control rate (DCR) by adding patients with stable disease (SD) while receiving the CAPTEM regimen to those who were scored as having responses. PFS was determined as the date of progression, death from any cause, or the last follow-up for patients without progression. The follow-up period was defined as the time from disease diagnosis to the last follow-up or date of death. Overall survival (OS) was calculated as the time from the initiation of CAPTEM to either the date of the last follow-up or the date of death. Patients who received at least one cycle of CAPTEM were included in the PFS and OS analyses. Patients who received modified doses were included in the study. Adverse events were extracted from the patient files. The type and severity of the adverse event were recorded in patient files by the treating physician at each center in accordance with the CTCAE-grading system (CTCAE version 4.0).

Ethics committee approval was obtained from Istanbul Bilgi University (Project number: 2023-40162-052).

### Statistical Analysis

Data were analyzed using the SPSS 22.0 software. Chi-square analysis was performed to compare the demographic and clinicopathological characteristics of the patients across all 3 groups. It was also used to compare treatment responses among all groups. The Kaplan-Meier method was utilized to estimate the median PFS and OS values of the patients, and to compare these findings according to treatment line, histological grade, Ki-67 levels, and primary tumor localization. Cox regression analysis was used to investigate the factors influencing median PFS and OS in the entire patient population. In all tests, a *P*-value of less than .05 was considered to be statistically significant.

## Results

A total of 308 patients with metastatic NETs who were administered CAPTEM between June 2010 and December 2022 were included in the study. The median follow-up time was 41.0 months (range: 1.7-212.1 months), and the median age was 53 years (range: 22-79 years). The clinicopathological characteristics of all patients and those who received CAPTEM regimen as first-, second-, or ≥third-line treatment is shown in Table 1.

**Table 1.** Patient characteristics.

Number of the patients	All patients, *n* (%)	First line, *n* (%)	Second line, *n* (%)	≥Third line, *n* (%)	*P*-value
Number	308 (100)	139 (45.1)	118 (38.3)	51 (16.6)	
Age median (min-max)	53 (22-79)	52 (18-82)	57 (18-80)	55 (19-80)	
Age groups					.39
<50	124 (44.6)	60 (43.2)	44 (37.3)	20 (39.2)	
50-70	154 (42)	66 (47.5)	5 (50)	29 (56.9)	
>70	30 (13.4)	13 (9.4)	15 (12.7)	2 (3.9)	
Gender					.24
Female	151 (49)	61 (43.9)	64 (54.2)	26 (51)	
Male	157 (51)	78 (56.1)	54 (45.8)	2 (49)	
PS (ECOG)					.53
0	155 (50.3)	76 (54.7)	58 (49.2)	21 (41.2)	
1	118 (38.3)	50 (36.0)	45 (38.1)	23 (45.1)	
2	35 (11.4)	13 (9.4)	15 (12.7)	7 (13.7)	
Comorbidities^*^					.53
Yes	137 (44.5)	64 (46.0)	48 (40.7)	25 (40)	
No	171 (55.5)	75 (54.0)	70 (59.3)	26 (51)	
Site (primary)					.39
Pancreas	145 (47.1)	75 (54.0)	48 (40.7)	22 (43.1)	
Gastrointestinal system	90 (29.2)	34 (24.5)	39 (33.1)	17 (33.3)	
Lung	57 (18.5)	22 (15.8)	26 (22)	9 (17.6)	
Unknown	16 (5.2)	8 (5.8)	5 (4.2)	3 (5.9)	
Histologic grade					**.01**
Grade 1	62 (20.1)	19 (13.7)	33 (28.0)	10 (19.6)	
Grade 2	193 (62.7)	104 (74.8)	65 (55.1)	24 (47.1)	
Grade 3	40 (13)	14 (10.1)	11 (9.3)	15 (29.4)	
Unknown (Ki-67 1-20%)	13 (4.2)	2 (1.4)	9 (7.6)	2 (3.9)	
Metastatic sites					.34
Liver	101 (32.8)	37 (26.6)	45 (38.1)	19 (37.3)	
Extra-liver	54 (17.5)	26 (18.7)	20 (16.9)	8 (15.7)	
Liver and extra-liver	153 (49.7)	76 (45.1)	53 (44.9)	24 (47.1)	
Ki-67 (%)					.27
<3	63 (20.5)	27 (19.4)	33 (23.7)	8 (15.7)	
3-20	196 (63.6)	91 (65.5)	76 (64.4)	29 (56.9)	
21-55	37 (12)	16 (11.5)	10 (8.5)	11 (21.6)	
Unknown (grades 1/2)	12 (3.9)	5 (3.6)	4 (3.4)	3 (5.9)	
No. of cycles					
Median (min-max)	6 (1-64)	6 (1-64)	6 (1-39)	6 (2-21)	
Mean ± SD	8.21 ± 7.49	8.79 ± 8.13	8.12 ± 7.64	6.00 ± 4.82	
Prior surgery (curative)					.28
Yes	78 (25.3)	41 (29.5)	27 (27.9)	10 (19.6)	
No	230 (74.7)	98 (70.5)	91 (77.1)	41 (80.4)	
Previous treatment at metastatic stage, *n* (%)					
Somatostatin analog	106 (34.5)	**—**	73 (61.9)	33 (64.7)	
PRRT	27 (8.8)	**—**	10 (8.5)	17 (33.3)	
Cytotoxic chemotherapy	61 (19.8)	**—**	31 (26.3	30 (58.8)	
Everolimus	28 (9.1)	**—**	6 (5.1)	22 (43.1)	
Sunitinib	9 (2.9)	**—**	1 (0.8)	8 (15.7)	
Locoregional therapy	30 (9.7)	**—**	21 (17.8)	9 (17.6)	
Grade 3-4 adverse effects					.29
Yes	69 (22.4)	32 (23)	22 (18.6)	15 (29.4)	
No	239 (77.6)	107 (77)	96 (81.4)	36 (70.6)	
Drug holiday					.19
Yes	5 (16.9)	21 (15.1)	18 (15.3)	13 (25.5)	
No	256 (83.1)	118 (84.9)	100 (84.7)	38 (74.5)	
Dose reduction					**.001**
Yes	65 (21.1)	25 (18)	19 (16.1)	21 (41.2)	
No	243 (78.9)	114 (82)	99 (83.9)	30 (58.8)	
Treatment interruption					.37
Yes	23 (9.1)	8 (5.8)	9 (7.6)	6 (11.8)	
No	285 (92.5)	131 (94.2)	109 (92.4)	45 (88.2)	

Bold values indicate statistically significant (*P*<0.05).

^*^Hypertension, diabetes, coronary heart disease, chronic kidney disease, cerebrovascular disease, chronic obstructive pulmonary disease, thyroiditis.

**Figure 1.**
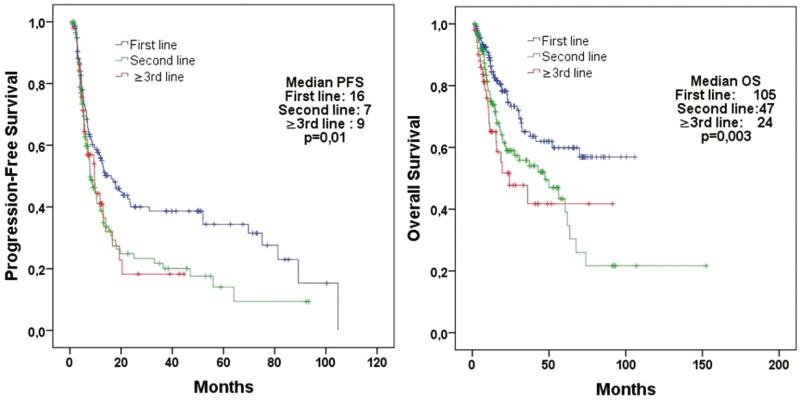
Progression-free survival according to the line of treatment (first-line therapy, *n* = 139; second-line therapy, *n* = 118; ≥third-line therapy, *n* = 51 patients).

The median PFS for all patients was 10.6 months, and the median OS was 60.4 months (Supplementary Fig. S1). The patients who received CAPTEM as first-, second-, or ≥third-line of treatment had median PFS values of 16.1, 7.9, and 9.6 months, respectively (*P* = .01), and median OS values of 105.8, 47.2, and 24.1 months, respectively (*P* = .003; Fig. 1).

The ORR of entire cohort was 42.2%, while the DCR was 68.1%. The patients who received CAPTEM as first-, second-, or ≥third-line had an ORR of 54.7%, 33.3%, and 30.0% (*P* < .001), respectively, and DCR of 71.5%, 65.0%, and 66.0%, respectively (*P* = .43; Table 2).

**Table 2.** Best responses according to the line of treatment.

Response category, *n* (%)	All patients (*n* = 304)^*^; n (%)	First-line therapy, (*n* = 137); *n* (%)	Second-line, therapy (*n* = 117); *n* (%)	≥ Third-line therapy, (*n* = 50); *n* (%)	*P*-value
Complete response (CR)	11 (3.6)	8 (5.8)	3 (2.6)	0 (0)	.004
Partial response (PR)	118 (38.8)	67 (48.9)	36 (30.8)	15 (30.0)	
Stable disease (SD)	78 (25.7)	23 (16.8)	37 (31.6)	18 (36.0)	
Progressive disease (PD)	97 (31.9))	39 (28.5)	41 (35.0)	17 (34.0)	
Objective response rarte (ORR) (CR + PR)	129 (42.4)	75 (54.7)	39 (33.3)	15 (30.0)	<.001
Disease control rate (DCR = CR + PR + SD)	207 (68.1)	98 (71.5)	77 (65.0%)	33 (66.0)	.43

^*^Four patients given only one course due to side effects were not included in the response rate analysis.

**Figure 2.**
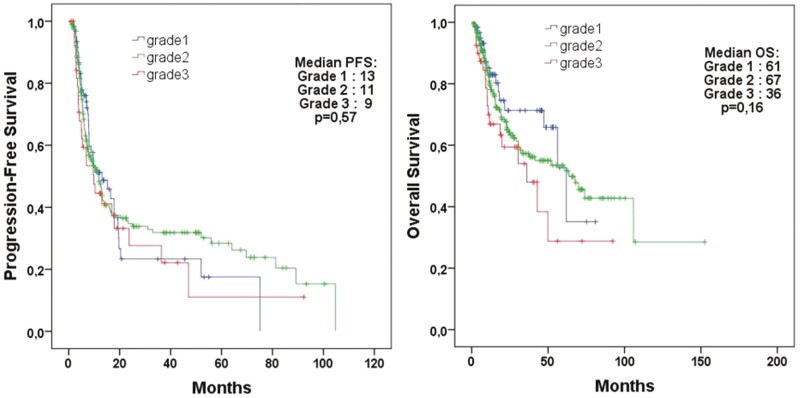
Survival analysis of all patients treated with CAPTEM treatment according to grade.

The median PFS was 13.1 months for patients with grade 1 tumors, 11.9 months for patients with grade 2 tumors, and 9.8 months for patients with grade 3 tumors (*P* = .57; Fig. 2). The median OS was 61.9, 67.6, and 36.0 months, respectively (*P* = .16).

**Figure 3.**
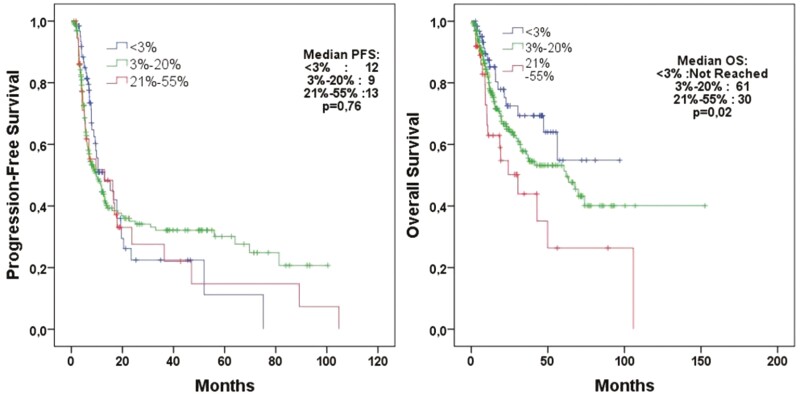
Progression-free survival according to the Ki-67 groups (<3%, 3%-20%, 20%-55%) (*n* = 308).

When evaluating patients according to the Ki-67 status of their tumors in the PFS analysis, median PFS values were 12.9, 9.8, and 13.0 months for patient whose tumors had Ki-67 values of <3%, 3%-20%, and >20%, respectively (*P* = .76; Fig. 3). Median OS values were not reached, 61.9 months, and 30.4 months for patient whose tumors had Ki-67 values of <3%, 3%-20%, and >20%, respectively (*P* = .02).

The Cox regression analysis that examined the factors affecting PFS showed that first-line treatment, and prior curative surgery were statistically significant factors. In the Cox regression analysis for OS, first-line treatment, Ki-67 level of 21%-55%, previous curative surgery, age ≤50, and ECOG PS 0-1 were identified as statistically significant factors (Table 3).

**Table 3.** Cox regression analysis of variables associated with PFS and OS in the 308 patients treated with CAPTEM.

	Progression-free survival			Overall survival		
	HR	95% CI	*P* value	HR	95% CI	*P* value
Gender (male vs. female)	0.91	0.79-1.05	.22	0.84	0.69-1.01	.07
Age (≤50 vs. > 50)	1.17	0.87-1.57	.29	1.99	1.32-3.00	**.001**
Comorbidities (yes vs. no)	1.09	0.94-1.27	.21	0.92	0.76-1.10	.37
PS (ECOG 0-1 vs. 2)	0.97	0.77-1.21	.79	0.75	0.58-0.97	**.03**
Site type (pancreas vs. others)	0.88	0.66-1.17	.39	0.87	0.60-1.26	.46
Ki- 67 (≤20% vs. 21%-55%)	1.15	0.75-1.75	.51	1.80	1.09-2.96	**.02**
Grade (1-2 vs. 3)	1.24	0.81-1.88	.30	1.54	0.92-2.57	.09
First-line therapy vs. subsequent therapies^*^	0.80	0.69-0.93	**.003**	0.72	0.60-0.88	**.001**
Prior surgery (curative)	1.21	1.02-1.44	**.02**	1.48	1.14-1.90	**.002**

Bold values indicate statistically significant (*P*<0.05).

^*^Subsequent therapies include second “,third and more lines therapies”.

Grade 3-4 side effects were observed in 69 patients (21.1%). Dose reduction was implemented in 65 patients (16.9%). The rate of dose reduction was higher in patients receiving treatment in ≥third line (41.6%) compared to those receiving treatment in first line (18.0%) or second line (16.1%; *P* = .001; Tables 1 and 4). The most common side effects that led to treatment discontinuation were gastrointestinal and hematological side effects. Treatment was discontinued in 4 patients due to the development of febrile neutropenia (Table 4). None of the patients were continued on a single drug as maintenance therapy. There were no treatment-related deaths. Data on the duration and recovery of cytopenias were not collected.

**Table 4.** Adverse-effects of CAPTEM chemotherapy (*n* = 308).

	Grade 1, 2 *n* (%)	Grade 3, *n* (%)	Grade 4, *n* (%)	Treatment interruption, *n* (%)
Decreased platelet count	34 (11.0)	8 (2.6)	3 (1.0)	3 (1.0)
Decreased neutrophil count	34 (11.0)	1 (0.3)	3 (1.0)	4 (1.3)
Decreased lymphocyte count	40 (12.9)	3 (1.0)	—	
Anemia	85 (27.6)	10 (3.2)	—	
Fatigue	127 (41.3)	10 (3.2)	1 (1.2)	3 (1.0)
Nausea/vomiting	116 (37.7)	14 (4.5)	—	5 (1.6)
Constipation	11 (3.5)	1 (0.3)	—	
Gastrointestinal hemorrhage	4 (1.3)	2 (0.6)	—	2 (0.6)
Diarrhea	34 (11.0)	3 (1.0)	2 (0.6)	2 (0.6)
Anorexia	91 (29.6)	4 (1.3)	1 (0.3)	1 (1.2)
Abdominal pain	27 (8.8)	2 (0.6)	1 (0.3)	2 (0.6)
Fever	4 (6.8)	—	—	
Oral mucositis	42 (13.6)	4 (1.3)	—	2 (0.6)
Pruritis	4 (6.8)	—	—	
Skin rash	2 (3.4)	—	—	
Palmar-plantar erythrodysesthesia	58 (18.8)	4 (1.3)	—	1 (1.2)
Increased blood bilirubin	15 (4.8)	5 (1.6)	1 (0.3)	1 (1.2)
Increased alkaline phosphatase	22 (7.1)	4 (1.3)	—	
Increased AST	41 (13.3)	2 (0.6)	—	1 (1.2)
Increased ALT	32 (10.4)	1 (0.3)	—	1 (1.2)
Dehydration	2 (2.4)	2 (2.4)	—	

When evaluating patients according to primary tumor location, median PFS was found to be 9.8 months for the pancreas, 12.7 months for the gastrointestinal system (GIS), 17.8 months for the lung, and 6.4 months for unknown primary site; there was no significant difference in median PFS according to the primary tumor site (*P* = .45). Similarly, for OS, there was no significant difference between pancreas (47.2 months), GIS (56.1 months), lung (73.9 months), and unknown primary site (27.1 months) (*P* = .85; Supplementary Fig. S2).

## Discussion

Our study is a retrospective, multicenter analysis involving 34 centers and to the best of our knowledge represents the largest cohort to date examining the use of the CAPTEM regimen in patients with metastatic neuroendocrine tumors (NETs). Importantly, our study is unique with stratification of patients based on the line of treatment. Previous studies analyzing the CAPTEM regimen have struggled with the inclusion of more heterogenous patient populations, with differing grades and Ki-67 levels, and the inclusion of patients with NEC in some studies.^4,16,17^ By excluding patients with NEC, we aimed to create a more homogeneous group of 308 patients with diagnoses of metastatic NETs, recognizing some of our patients with pathologic grades 1 and 2 tumors had levels of Ki67 as high as 55%. We found a median PFS of 10.6 months and a median overall survival (OS) of 60.4 months for all patients. When stratified by the line of treatment, the median PFS was 16.1, 7.9, and 9.6 months for CAPTEM administered as first-, second-, and ≥third-line treatment, respectively (*P* = .01), and the median OS was 105.8, 47.2, and 24.1 months, respectively (*P* = .003). Notably, CAPTEM administered as first-line treatment significantly improved both PFS and OS compared to other lines of treatment. Furthermore, CAPTEM administered as first-line treatment yielded a higher ORR than other regimens (54.7%, 33.3%, and 30.0%, respectively, *P* < .001). These findings suggest that first-line CAPTEM, which demonstrated superiority in terms of median PFS, OS, and ORR, is more effective than later lines of treatment. However, we cannot exclude an impact of the concurrently administered somatostatin analogs, given these agents were shown to improve PFS, but not OS due to their antiproliferative effects as reported for lanreotide in CLARINET and for octreotide in PROMID.^18,19^ The discontinuation rate for CAPTEM was 9.5%, and grade 3-4 side effects were observed in 22.5% of the patients, indicating that CAPTEM is a well-tolerated treatment regimen. There was no significant difference in the side effect profile between patients receiving first line and other lines of treatment (Table 1).

In a meta-analysis of 42 studies with 1818 patients diagnosed with NENs and treated with CAPTEM, the ORR was 37.1%, and the DCR was 77% (SD: 40%, PR: 34.8%, CR: 2.3%, progressive disease, PD: 18.5%). This meta-analysis also reported that the median PFS ranged from 4 to 38 months and the median OS ranged from 8 to 103 months.^20^ A study by Chatezellis et al that involved 79 patients found a median PFS of 10.1 months, similar to our study, but a higher OS (102.9 months) than our study.^7^ A recently reported randomized, phase II trial in advanced low-grade or intermediate-grade pancreatic NETs compared temozolomide versus capecitabine/temozolomide in 133 patients, finding a median PFS of 14.4 months for temozolomide versus 22.7 months for capecitabine/temozolomide (hazard ratio = 0.58), but no difference in overall survival, 53.8 versus 58.7, respectively (*P* = .42).^14^

Our results, consistent with the literature, show that first-line CAPTEM treatment is more effective than other lines.^21-25^ Strosberg et al found a median PFS of 18 months and an ORR of 70% with first-line CAPTEM treatment.^21^ In a study by Liu et al, the median PFS was 10.3 months for first-line CAPTEM treatment versus 4.4 months for second-line treatment in patients with high-grade metastatic NETs; the ORR was 50% versus 20%, suggesting first-line treatment was more effective.^22^ The study of Crespo et al that involved 65 patients diagnosed with multicentric grade 1-2 NETs showed that first-line CAPTEM treatment offered a higher median PFS and OS benefit compared to other lines.^23^ In the study by Peixoto et al, when CAPTEM treatment was given as the first-line treatment to patients with pancreatic NET, the median PFS was 15.9 months versus 3.2 months for other lines (*P* = .04).^24^ In addition, similar findings in the study by Sahu et al, suggest a potentially better treatment response and survival advantage for grade 3 patients with neuroendocrine tumors receiving CAPTEM as first-line treatment versus ­second-line.^25^ In our study, we also observed a contribution of first-line treatment to PFS, OS, and ORR (Fig. 1and Table 2). Moreover, in our Cox regression analysis, first-line CAPTEM treatment was identified as a significant factor influencing both PFS and OS compared to other lines (for PFS, HR: 0.80, *P* = .003, 95% CI, 0.69-0.93; and for OS, HR: 0.72, *P* = .001, 95% CI, 0.60-0.88). In the study by Wang et al, first-line treatment was determined as one of the factors affecting PFS in patients with neuroendocrine tumors with a Ki-67 index of 10%-40%.^26^ Although not statistically significant in the phase II study by Jeong et al in patients with grade 3 gastroenteropancreatic NETs and Ki-67 < 55%, first-line treatment was observed to significantly contribute to PFS (16.5 vs. 4.6 months, *P* = .42) compared to other lines.^27^ Furthermore, in a study that was performed in 3 cancer centers, the median PFS was 24.4 months for first-line treatment, 7.1 months for ­second-line treatment, 10.7 months for third-line treatment, and 11.1 months for fourth-line treatment. Although this difference was not statistically significant, a notable PFS advantage was observed in the first-line arm (*P* = .372).^18^

In evaluating patients with neuroendocrine tumors categorized based on tumor grade and Ki-67 index levels (<3%, 3%-20%, 21%-55%), we observed no difference in PFS across the groups. However, there was a significant difference in overall survival (OS) as illustrated in Figs. 2 and 3. A study by Al-Tobuah et al with 462 patients with metastatic NEN found a similar result: there was no difference in PFS between patients with grades 1, 2, and 3 NENs, but there was a significant difference in terms of OS.^11^ No statistically significant difference in PFS was observed between patients with grades 2 and 3 NENs in the study by Sahu et al (10 vs. 5 months, *P* = .3).^25^ In a study that included 143 patients with metastatic pancreatic NETs by Cives et al, tumor grade, Ki-67 index, or mitotic rate was among the factors affecting PFS and OS.^28^ This might be due to the fact that the cytotoxic activity of temozolomide is not confined to mitosis but spans the whole cell cycle.^29^ It also underscores the idea that tumor proliferative activity, as measured on needle biopsy or resected primary tumor specimen, may not always reflect the clinical aggressiveness of metastatic NETs.^29^ Al-Tobuah et al, in a study with 32 patients diagnosed with midgut NET, found no significant difference in median PFS between patients with grades 1, 2, and 3 tumors and surprisingly, higher PFS in patients with high-grade tumors (median PFS 10 vs. 40, *P* = .176; median OS 58 versus 140, *P* = .093).^30^ In our study, when we grouped the Ki-67 index as ≤20% versus 21-55%, no significant difference was found in PFS. There is no established Ki-67 cutoff value for recommending chemotherapy.^31^ Temozolomide-based chemotherapy is preferably used in pancreatic G3 NET or in gastrointestinal NEC with a Ki-67 index of <55%.^32,33^ In a receiver operating characteristic (ROC) curve analysis for ORR in a study by Chatzellis et al that involved 143 patients with advanced NEN, Ki-67 level was found as a poor predictor of treatment response (AUC = 0.678), and it was reported Ki-67 level was not a factor affecting PFS.^7^ Similar to our results, this study also found that curative surgery was one of the factors affecting both PFS and OS.^7^ In our cohort, we subdivided 308 patients into groups according to the primary tumor location (pancreas, gastrointestinal tract, lung, and unknown). We observed no significant difference in median PFS and OS between these groups (Supplementary Fig. S2). In addition, when we grouped the primary tumor site as pancreatic and non-pancreatic, it was not found to be a factor influencing PFS and OS (Table 3). A multicenter study by Crespo et al also reported no difference in PFS and OS when considering the location of the primary tumor as pancreas NET or non-pancreas NET.^23^ Studies by Peixoto et al and Wang et al also showed no significant differences in ORR and PFS between patients with or without pancreatic origin.^31,33^ However, studies by Al-Tobuah et al and Thomas et al reported better PFS and OS in patients with a ­pancreatic-origin of their tumors receiving CAPTEM treatment compared to ­non-pancreatic origin tumors ^24,26^.

In our study, 69 patients (22.4%) discontinued the CAPTEM regimen due to grade 3-4 side effects, and 23 patients (9.1%) due to side effects. A meta-analysis on CAPTEM reported grade 3-4 side effects in 16.4% of patients across 29 studies.^19^ As per the literature, CAPTEM therapy is a safe combination with tolerable side effects.^20-31^ In our study, no significant difference was observed in terms of side effects when comparing the different lines of treatment (Tables 1 and 4).

The main limitation of our study is its retrospective design, which carries the potential for selection bias. However, given the rarity of NETs, it is challenging to conduct prospective studies. The data from these nonrandomized studies make it difficult to derive specific treatment recommendations. Nonetheless, we feel that our multicenter study involving 34 centers can contribute meaningful results to the literature. Another limitation we should mention is the data on progression were retrieved from the radiology reports rather than reanalyzed images for the study due to personal data restriction regulations. It is noteworthy that although clinical guidelines do not recommend CAPTEM as first-line treatment for NENs, it was used as first-line treatment in 45.1% of our patients. The best response to treatment was retrieved from the available radiology reports, and no additional measurement was conducted for the study. Another potential weakness of this current study is the lack of assessment of MGMT expression.

## Conclusion

Our large, multicenter, retrospective cohort study of patients with metastatic NET found that first-line CAPTEM treatment yielded higher ORR, PFS, and OS benefits compared to other lines of treatment regardless of the tumor location. This therapy is administered safely in our daily practice to patients with tumors arising from both the pancreas and other sites, demonstrating that it is a tolerable combination regimen. To substantiate our findings, evaluation of CAPTEM treatment through prospective phase III randomized trials is required.

## Supplementary Material

oyad257_suppl_Supplementary_Figure_S1

oyad257_suppl_Supplementary_Figure_S2

oyad257_suppl_Supplementary_Figure_Captions

## Data Availability

The data underlying this article will be shared on reasonable request to the corresponding author.
