# Supplementary figures and images for: Efficacy of Capecitabine and Temozolomide Regimen in Neuroendocrine Tumors: Data From the Turkish Oncology Group
Source: Oncologist. 2023 Sep 7;28(10):875–84. doi: 10.1093/oncolo/oyad257 (PMC10546829; doi:10.1093/oncolo/oyad257)

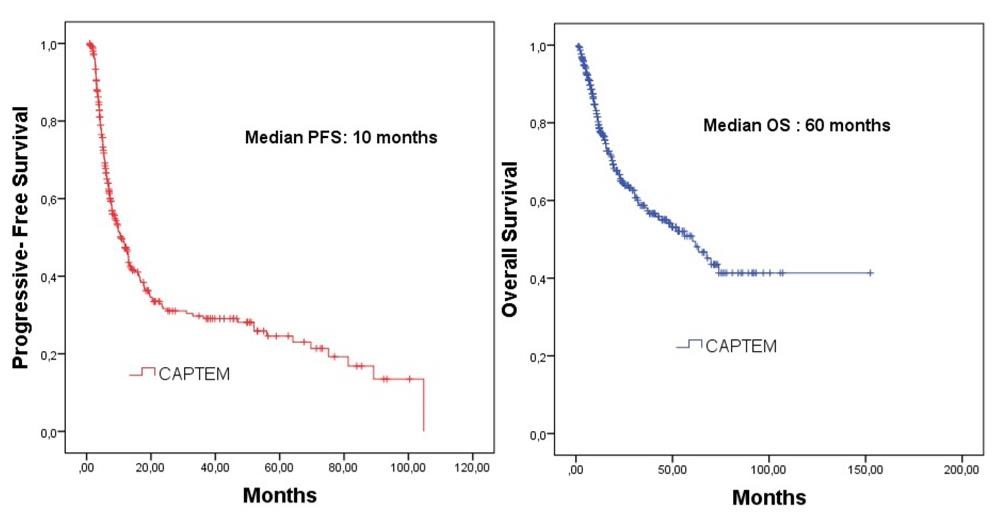

Supplement: oyad257_suppl_Supplementary_Figure_S1 [file oyad257_suppl_supplementary_figure_s1.jpeg]

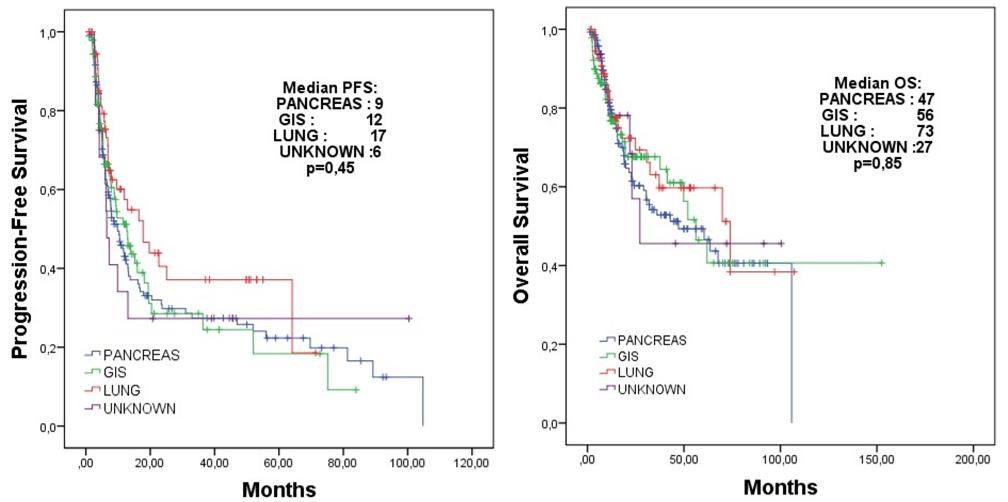

Supplement: oyad257_suppl_Supplementary_Figure_S2 [file oyad257_suppl_supplementary_figure_s2.jpeg]
