# Supplementary material for: Efficacy of Capecitabine and Temozolomide Regimen in Neuroendocrine Tumors: Data From the Turkish Oncology Group
Source: Oncologist. 2023 Sep 7;28(10):875–84. doi: 10.1093/oncolo/oyad257 (PMC10546829; doi:10.1093/oncolo/oyad257)
Supplement: oyad257_suppl_Supplementary_Figure_Captions [file oyad257_suppl_supplementary_figure_captions.docx]

**Supplementary Figure 1:** Survival analysis of all patients treated with CAPTEM treatment (n=308)

**Supplementary Figure 2:** Survival analysis according to the primary site (n=308)
